# Allies or Enemies: The Role of Reactive Oxygen Species in Developmental Processes of Black Cottonwood (*Populus trichocarpa*)

**DOI:** 10.3390/antiox9030199

**Published:** 2020-02-27

**Authors:** Katarzyna Marzec-Schmidt, Natalia Wojciechowska, Klaudia Nemeczek, Agnieszka Ludwików, Joanna Mucha, Agnieszka Bagniewska-Zadworna

**Affiliations:** 1 Department of General Botany, Faculty of Biology, Institute of Experimental Biology, Adam Mickiewicz University, Uniwersytetu Poznańskiego 6, 61-614 Poznań, Poland; natalia.wojciechowska@amu.edu.pl (N.W.); klaudia.wypychowska.030393@gmail.com (K.N.); agabag@amu.edu.pl (A.B.-Z.); 2 Department of Biotechnology, Faculty of Biology, Institute of Molecular Biology and Biotechnology, Adam Mickiewicz University, Uniwersytetu Poznańskiego 6, 61-614 Poznań, Poland; ludwika@amu.edu.pl; 3 Laboratory of Ecology, Institute of Dendrology, Polish Academy of Science, Parkowa 5, 62-035 Kórnik, Poland; jmucha@man.poznan.pl

**Keywords:** antioxidants, microarrays, programmed cell death, *Populus trichocarpa*, reactive oxygen species, senescence, xylogenesis

## Abstract

In contrast to aboveground organs (stems and leaves), developmental events and their regulation in underground organs, such as pioneer and fine roots, are quite poorly understood. The objective of the current study was to achieve a better understanding of the physiological and molecular role of reactive oxygen species (ROS) and ROS-related enzymes in the process of stem and pioneer root development in black cottonwood (*Populus trichocarpa*), as well as in the senescence of leaves and fine roots. Results of a transcriptomic analysis revealed that primary/secondary growth and senescence are accompanied by substantial changes in the expression of genes related to oxidative stress metabolism. We observed that some mechanisms common for above- and under-ground organs, e.g., the expression of superoxide dismutase (SOD) genes and SOD activity, declined during stems’ and pioneer roots’ development. Moreover, the localization of hydrogen peroxide (H_2_O_2_) and superoxide (O_2_•^–^) in the primary and secondary xylem of stems and pioneer roots confirms their involvement in xylem cell wall lignification and the induction of programmed cell death (PCD). H_2_O_2_ and O_2_•^–^ in senescing fine roots were present in the same locations as demonstrated previously for ATG8 (AuTophaGy-related) proteins, implying their participation in cell degradation during senescence, while O_2_•^–^ in older leaves was also localized similarly to ATG8 in chloroplasts, suggesting their role in chlorophagy. ROS and ROS-related enzymes play an integral role in the lignification of xylem cell walls in *Populus trichocarpa,* as well as the induction of PCD during xylogenesis and senescence.

## 1. Introduction

Reactive Oxygen Species (ROS) are highly reactive chemical compounds containing oxygen in their structure [1], which affect oxygen homeostasis when cells are exposed to environmental stress conditions and are responsible for oxidative damage to cells and even cell death [2]. ROS, however, also play an important role in many developmental processes in plants, acting as signalling molecules [3,4].

Antioxidants function in both alleviating oxidative stress and the generation of ROS. The ability of antioxidants to scavenge ROS and maintain a balance between the production and degradation of ROS is crucial for normal plant function and development [5]. Elevated levels of ROS also play a role in plant development, in processes such as programmed cell death (PCD) [6]. PCD may seem disordered and injurious, but it is a strictly regulated and well-organized process. PCD induces physiological and molecular responses that are an inseparable element of the proper functioning and homeostasis of living organisms. PCD is dependent on the presence of many molecules within an organism [7], including elevated levels of ROS. ROS act as signalling molecules that trigger a cascade of events regulating developmental processes, such as the formation of xylary elements, including fibres, tracheids, and vessels [8,9]. ROS also play a role in the regulation of leaf senescence, where they function as signal molecules that trigger a string of events leading to cell death [10]. The role of hydrogen peroxide (H_2_O_2_) in xylogenesis is well known [11], where it is produced in vascular precursor cells generated by the cambium. When primary xylem is formed and cell walls are lignified, production of H_2_O_2_ continues and accumulates at a relatively high level [8,12]. Excessive accumulation of H_2_O_2_ above optimal levels oxidizes peroxidases [8], which act as a signal to initiate a cascade of successive events, including the rupture of the vacuolar membrane, cytoplasmic degradation, and damage to the nucleus. These events were confirmed in studies conducted on *Eucommia ulmoides,* where high levels of DNA degradation were demonstrated during PCD events occurring in xylogenesis [13]. All cellular structures are lysed, and the protoplast is degraded during the final stage of PCD-regulated xylogenesis [14]. As a result, tracheary elements (TEs) are created which function to conduct water and minerals throughout a plant. It was demonstrated that the accumulation of hydrogen peroxide increases the expression of a gene encoding metacaspase 8, which participates in the PCD during xylogenesis. H_2_O_2_ acts as a signaling molecule and triggers further cell differentiation [15]. In the final stage of xylogenesis, when mature proto- and metaxylem with strongly lignified cell walls are formed, hydrogen peroxide is no longer produced [12]. Changes in hydrogen peroxide levels do not directly cause cell death but rather trigger a signal transduction cascade that leads to cell degradation [16,17].

In addition to the role of ROS in xylogenesis, they also play a crucial role in senescence, which is the final stage of development. Senescence is a genetically programmed sequence of events that represents the last stage of plant development [18]. Despite its destructive character, senescence is also a precisely regulated process controlled by many factors. One of the earliest steps in the senescence process is the generation of ROS [19]. At this step, the ROS generation and accumulation is associated with a disruption in the balance between the production of antioxidant enzymes, such as catalase (CAT) and ascorbate peroxidase (APX), and the excess accumulation of ROS [20]. Similar to its role in xylogenesis, ROS acts as a signal molecule during senescence, however, high concentrations of ROS can induce oxidative stress and injure cells [21]. 

ROS are viewed as the initiators of protein degradation in chloroplasts and these organelles are also the main source of ROS generation during leaf senescence [22]. In addition, ROS are also responsible for the degradation of cell membranes due to lipid peroxidation. Transgenic approaches have been used to provide evidence pertaining to the role of ROS during the molecular regulation of leaf senescence [23,24,25] and oxidative stress tolerance has been linked to leaf longevity [25].

In addition to leaf senescence, ROS influences flower petal senescence [26]. The concentration of ROS in petals increases as visible symptoms of senescence become apparent [27]. This relationship was demonstrated in both an ethylene-sensitive species, such as carnation [28], as well as in an ethylene-insensitive species, such as lily [29]. The main sources of ROS production in flower petals are peroxisomes, mitochondria, and the apoplast [30]. While the role of ROS in the senescence of leaves and flower petals is relatively well known, there is still insufficient knowledge regarding the role of ROS in the senescence of fine roots.

While the involvement of ROS in developmental processes in aboveground organs, including stems, leaves, and flower petals, has been widely studied, little is known about the role of ROS in the development and senescence of underground organs, such as pioneer and fine absorptive roots. Therefore, the present study focused on developing a better understanding of the complex role of ROS and ROS-related enzymes in the process of the primary and secondary growth of pioneer roots compared to stems in black cottonwood (*Populus trichocarpa*). In addition, the role of ROS in the senescence of fine roots was also compared to leaves. A microarray analysis was conducted to identify ROS-related genes involved in primary and secondary growth and senescence to determine whether common mechanisms exist for these processes in aboveground and underground organs of *Populus trichocarpa*. No such comparative information on this topic has been previously reported. Results of the transcriptomic analysis of pioneer roots and fine roots provide new insights into the role of ROS and ROS-related enzymes in the development and senescence of these understudied organs. Tissue localization of superoxide and hydrogen peroxide and measurement of superoxide dismutase activity (which converts superoxide to hydrogen peroxide) was also conducted to better understand the role of ROS in plant development. A comprehensive characterization of different plant organs at different developmental stages (from tissue differentiation to senescence) will address the question: Are ROS allies or enemies of cells and tissue during plant ontogeny? This conceptual question has not been addressed in the literature, especially for underground organs. 

## 2. Materials and Methods 

### 2.1. Plant Material and Growth Conditions

All of the analyses were conducted on *Populus trichocarpa* (Torr. and A. Gray ex Hook.). Seeds were obtained commercially from the FLORPAK Seeds Store, Młynki, Poland. Seedlings were initially grown in a plant growth chamber (Conviron GR96, Conviron, Winnipeg, MB, Canada) at 18/14 °C and a 16/8 h photoperiod. Plants were then transferred into rhizotrons after a three-month period. Roots were grown in clear-walled chambers filled with natural soil and shoots were grown normally, as described by Wojciechowska et al. [18,31].

For the analyses, stems were divided into segments corresponding to their developmental stage: 0–2 cm—apical meristem with primary growth (PS), 20–25 cm—secondary growth (SS), 40–45 cm—isolated secondary xylem (SX). Similarly, pioneer roots were also divided into similar segment categories: 0–2 cm—root tip with apical meristem (RT), 4–6 cm—primary growth (PR), 13–16 cm—secondary growth (SR). With regard to the sampling for different stages of senescence, fine roots and leaves were collected at three different times during the growing season. Leaves and fine roots were first sampled in early summer (7–15 July), when they were fully developed and functional, and were considered as leaf (LC) and fine root (RC) control samples. The second sampling of leaves and roots was done in early autumn (1–7 October) when levels of chlorophyll in leaves had declined by roughly 40% (LS1) and the colour of fine roots had changed from white to brown (RS1). The third collection was made during the middle of autumn (2–9 November), when chlorophyll levels in leaves had decreased by approximately 60% (LS2) and fine roots were dark brown or black in colour (RS2) (Table 1). Stem, pioneer root, leaf and fine root samples for each of three biological replicates were harvested from at least three individual plants. 

### 2.2. Microarray Analyses

Total RNA was extracted in triplicate from the plant samples described in the previous section using an RNeasy Plant Mini kit (Qiagen, Germantown, MD, USA). RNA quantity and quality were checked using a NanoDrop1000 (Thermo Fisher Scientific Inc., Waltham, MA, USA). cRNA synthesis and microarray hybridization to an Affymetrix GeneChip Poplar Genome Array (A-AFFY-131) were conducted according to the protocol described by the Affymetrix Laboratory of Microarray Analysis (Institute of Biochemistry and Biophysics Polish Academy of Science, Warsaw, Poland). The complete microarray dataset was submitted to the Gene Expression Omnibus database (accession number GSE126842 and GSE143559). All steps of the microarray data analysis were performed as previously described by Marzec-Schmidt et al. [32].

### 2.3. Activity of Superoxide Dismutase (SOD) Assay

The enzyme extract was prepared in triplicate from the pioneer root and stem samples described in the previous section. SOD activity was not determined in senescent leaves because differentially expressed SOD-encoding genes were not identified in the microarray analysis. In addition, SOD activity was not determined in fine roots due to difficulties in obtaining sufficient material for analysis, especially of older fine roots. Frozen pioneer root and stem samples (0.2 g) were ground to a fine powder in liquid N_2_ with a chilled mortar and pestle. The powder was then homogenized at 4 °C with 2 mL of cold 50 mM potassium-phosphate buffer (pH 7.0) containing 1 mM EDTA (ethylenediaminetetraacetic acid), 1% PVP (polyvinylpyrrolidone), 2% β-mercaptoethanol, and 0.1% Triton-X-100. The homogenate was centrifuged at 14,000 rpm at 4 °C. The resulting supernatant was collected and stored at –80 °C until further analysis. The activity of SOD was measured using the method described by Beauchamp and Fridovich [33]. The reaction mixture contained 0.1 M phosphate buffer (Ph 7.5) with 0.1 mM EDTA, 840 μM NBT, 150 mM methionine, and 2.4 μM riboflavin (8:1:1:1:1 by volume). Enzyme extract was added to the mixture in a proportion that inhibition of NBT oxidation occurred within a range of 20–80%. Absorbance was measured spectrophotometrically at λ = 550 nm (Hitachi Double Beam Spectrophotometer U-2900, Hitachi High-Technologies Co., Tokyo, Japan) after 20 min of incubation in the light (two 40 W light bulb) or in the darkness (a blank sample). SOD activity was expressed as units (U), where 1 U indicates the activity of enzyme causing the inhibition of NBT photoreduction to a blue formazan by 50% per 1 g of protein. SOD activity was measured in three biological replicates of each sample collection. Protein concentration was measured with a 2-D Quant Kit (GE Healthcare, Chicago, IL, USA) according to the manufacturer’s protocol. Statistical analyses (ANOVA and a Tukey’s test, *p* < 0.05) were performed using Statistica 12.0 software (StatSoft Poland Inc., Tulsa, OK, USA).

### 2.4. Localization of Superoxide

The modified method developed by Doke [34] and Ogawa et al. [35] was used to identify and localize superoxide anions in developing pioneer roots and stems, as well as in senescent leaves and fine roots. Nitro blue tetrazolium chloride (NBT) (pale yellow) forms a dark blue insoluble formazan dye in the presence of superoxide anion. Freshly sampled sections of stems, pioneer roots, leaves and fine roots were infiltrated with NBT buffer (0.05 g NBT) (Sigma-Aldrich, St. Louis, MO, USA) dissolved in 100 mL 2× phosphate-buffered saline PBS (Sigma-Aldrich, St. Louis, MO, USA) for 5 min using a vacuum pump (AGA LABOR, Warsaw, Poland). The plant material was further incubated with the buffer in the dark for 2 h and then rinsed with 80% ethanol. With the exception of fine roots, the plant material was sectioned (30 µm) using a vibratome (Leica VT 1200S, Leica Biosystems, Nussloch, Germany). Fine roots were fixed in a mixture of 2% (*v/v*) formaldehyde (Ph 6.8; Polysciences, Warrington, PA, USA) and 2% glutaraldehyde (pH 6.8; Polysciences, Warrington, PA, USA) for 12 h at 4 °C. The samples were subsequently rinsed three times in 1× PBS (Sigma-Aldrich, St. Louis, MO, USA) buffer to remove the fixative solution. Roots were dehydrated by transferring through a graded ethanol series (10–100%) and, after that, infiltrated and embedded in Paraplast Extra (melting point—57.8 °C; Sigma-Aldrich, St. Louis, MO, USA). Root were sectioned (20 μm) using a Leica RM2265 (Leica-Reichert, Bensheim, Germany) microtome. All sections were observed under a light microscope with an AxioCam MRc5 camera (Carl Zeiss, Jena, Germany) and the photographs were processed using AxioVision software (Carl Zeiss, Jena, Germany).

### 2.5. Localization of Hydrogen Peroxide 

The modified method of Thordal–Christensen et al. [36] was used to identify and localize hydrogen peroxide in stems, pioneer roots, leaves and fine roots. The dye, 3-3’ diaminobenzidine (DAB) (brown) forms an insoluble orange polymer in the presence of H_2_O_2_ and peroxidases. DAB (Sigma-Aldrich, St. Louis, MO, USA) was dissolved in HCl (1.2 mg 1 mL^−1^) to prepare the buffer and mixed with Tris in distilled water (0.1 mg 1 mL^−1^) in proportions to obtain a pH 3.8 solution. The plant material was infiltrated with the DAB buffer for 15 min using a vacuum pump (AGA LABOR, Warsaw, Poland). The material was further incubated in the buffer for 1 h in daylight and then rinsed with 80% ethanol which was preheated to 60 °C. With the exception of fine roots, the plant material was sectioned (30 µm) using a vibratome (Leica VT 1200S, Leica Biosystems, Nussloch, Germany). Fine roots were fixed in a mixture of 2% (*v/v*) formaldehyde (pH 6.8; Polysciences, Warrington, PA, USA) and 2% glutaraldehyde (pH 6.8; Polysciences, Warrington, PA, USA) for 12 h at 4 °C. The samples were subsequently rinsed three times in 1× PBS (Sigma-Aldrich, St. Louis, MO, USA) buffer to remove the fixative solution. Roots were dehydrated by transferring through a graded ethanol series (10–100%) and after that infiltrated and embedded in Paraplast Extra (melting point—57.8 °C; Sigma-Aldrich, St. Louis, MO, USA). Root were sectioned (20 μm) using a Leica RM2265 (Leica- Reichert, Bensheim, Germany) microtome. All sections were observed under a light microscope with an AxioCam MRc5 camera (Carl Zeiss, Jena, Germany) and the photographs were processed using AxioVision 4.9.1 software (Carl Zeiss, Jena, Germany).

## 3. Results

### 3.1. ROS and Oxidative Stress-Related Genes Are Overrepresented During the Primary/Secondary Growth of Stems and Pioneer Roots and Senescing Fine Roots and Leaves

Transcripts isolated from three developmental stages of poplar stems and pioneer roots were profiled to gain a comprehensive overview of gene expression during xylogenesis. Among the 56,055 targets present on the poplar microarray, 1171 targets were found to be differentially regulated in stems and 1914 in pioneer roots (one-way ANOVA, *p* ≤ 0.001, fold change ≥ 2). A detailed analysis of the results was previously described by Marzec-Schmidt et al. [32]. Transcripts isolated from two stages of senescing leaves and fine absorptive roots were also profiled to catalog gene expression during the process of senescence. Among the 56,055 targets, 1348 genes were differentially regulated in senescing leaves and 1898 in senescing fine roots (one-way ANOVA, *p* ≤ 0.001, fold change ≥ 2). The differentially expressed genes (DEGs) were subjected to a functional classification using the Database for Annotation, Visualization, and Integrated Discovery (DAVID) [37,38]. The most abundant functional categories in the DEGs of senescing fine roots were those related to ROS and oxidative stress. Notably, many ROS-related DEGs were also identified in developing stems and pioneer roots. Therefore, further analyses were conducted of the DEGs involved in ROS metabolism during xylogenesis in stems and pioneer roots, and of the DEGs associated with senescing fine roots and leaves.

#### 3.1.1. Primary and Secondary Growth of Plant Organs

##### Stems

**Figure 1:**
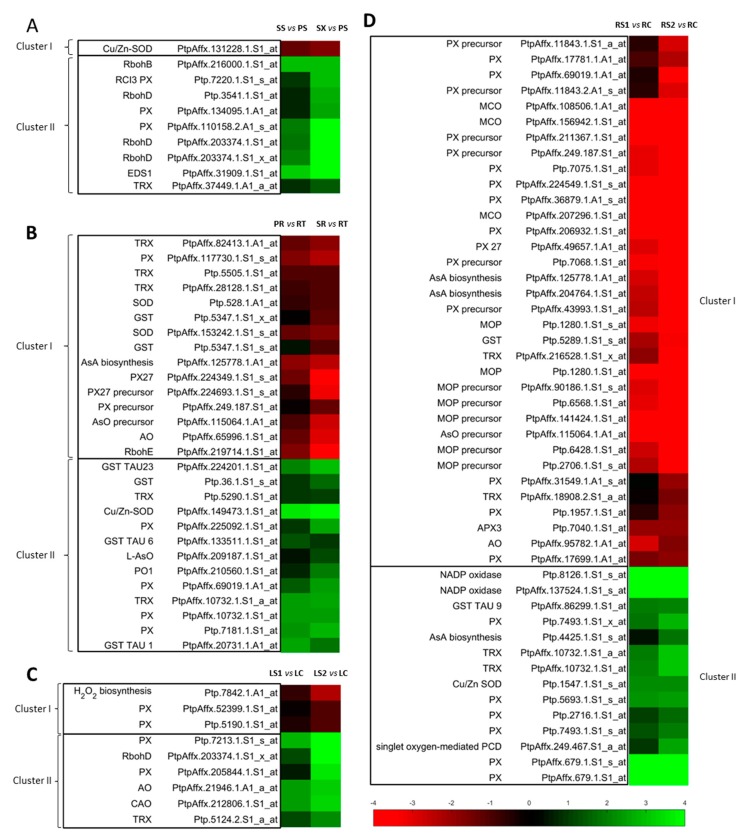
Heatmaps of the expression profiles of *Populus trichocarpa* genes related to reactive oxygen species (ROS) metabolism during primary and secondary growth of (**A**) stems and (**B**) pioneer roots, and during the senescence of (**C**) leaves and (**D**) fine roots.

DEGs related to ROS metabolism were mostly up-regulated and grouped in Cluster II, suggesting that they play a role in both primary and secondary xylogenesis. Only the gene encoding a poplar ortholog of Cu/Zn SOD 2 (PtpAffx.131228.1.S1_at) was down-regulated and grouped separately in Cluster I, potentially indicating its involvement in the early stage of xylogenesis (Figure 1A). 

##### Pioneer Roots

In contrast to the stem, most DEGs related to ROS metabolism were grouped in Cluster I and were down-regulated. Notably, however, genes encoding enzymes that play a crucial role in xylogenesis-related ROS metabolism were grouped in Cluster II. These included Cu/Zn SOD 1, thioredoxins (Ptp.5290.1.S1_at, PtpAffx.10732.1.S1_at, PtpAffx.10732.1.S1_a_at), peroxidases (PtpAffx.225092.1.S1_at, PtpAffx.69019.1.A1_at, Ptp.7181.1.S1_at), polyamine oxidase, glutathione S-transferases (Ptp.36.1.S1_s_at, PtpAffx.133511.1.S1_at, PtpAffx.224201.1.S1_at, PtpAffx.20731.1.A1_at), and L-ascorbate oxidase (Figure 1B).

#### 3.1.2. Senescing Plant Organs

##### Leaves

Most of the ROS-related DEGs (6) identified in senescing leaves were up-regulated and grouped in Cluster II, suggesting their potential role in early and late PCD signalling and its execution. Only three genes were down-regulated in the LS2 stage (Ptp.7842.1.A1_at, PtpAffx.52399.1.S1_at, Ptp.5190.1.S1_at) and clustered separately, indicating their potential involvement in the early signalling or prevention of PCD (Figure 1C).

##### Fine Roots

In contrast to senescing leaves, the majority of the 45 DEGs related to ROS metabolism were down-regulated and grouped in Cluster I. Among the 19 up-regulated DEGs in Cluster II were enzymes involved in ROS generation. These included NADP oxidases (Ptp.8126.1.S1_s_at, PtpAffx.137524.1.S1_s_at), Cu/Zn SOD, and PXs (Ptp.7493.1.S1_x_at, Ptp.5693.1.S1_s_at, Ptp.2716.1.S1_at, Ptp.7493.1.S1_s_at, PtpAffx.679.1.S1_s_at, PtpAffx.679.1.S1_at). Increasing ROS production and decreasing the synthesis of antioxidants in ageing fine roots would inhibit ROS scavenging and potentially promote PCD (Figure 1D). 

NADPH oxidases, which are also known as respiratory burst oxidase homologs (RBOHs), generate superoxide (O_2_•^–^) that spontaneously from hydrogen peroxide [39]. One gene encoding RBOHB and three *RBOHD* genes, all grouped in Cluster II, were identified in developing stems, and exhibited up-regulation during secondary stem growth (SS) and isolated xylem (SX) (Figure 1A). In contrast, only one gene encoding RBOHE was identified in pioneer roots and was down-regulated during development (Figure 1B). The up-regulation of genes encoding RBOHD was identified in senescing leaves (Figure 1C), and up-regulation of an NADP oxidase was observed in fine roots (Figure 1D).

Superoxide dismutases (SODs) convert O_2_• ^–^ into H_2_O_2_, which is a less harmful form of ROS [40]. Only one gene encoding Cu/Zn SOD2 exhibited altered expression during xylogenesis in stems and the transcript level of this gene decreased with the progression of stem development, suggesting that it played a functional role in processes occurring during early stem development (Figure 1A). Three genes encoding SOD were identified in pioneer roots. Two of them (PtpAffx.153242.1.S1_s_at and Ptp.528.1.A1_at) were down-regulated and grouped in Cluster I, while the third, encoding a Cu/Zn SOD1, was up-regulated in roots with primary (PR) and secondary (SR) growth (Figure 1B). Based on our results, SOD does not appear to be involved in the senescence process in leaves. It may, however, have a functional role in the senescence of fine roots, where increased expression of a Cu/Zn SOD gene was observed in the RS1 and RS2 stages of senescence (Figure 1D). 

*EDS1* (*enhanced disease susceptibility 1*) plays a crucial role in the generation and accumulation of H_2_O_2_ [41]. *ESD1* expression was strongly up-regulated during the secondary growth of stems (SS) and isolated secondary xylem (SX) (Figure 1A).

Peroxidases are involved in hydrogen peroxide production (hydroxylic and oxidative chemistry), as well as H_2_O_2_ scavenging (peroxidative chemistry) [40,42,43]. The three genes encoding peroxidases in stems grouped in Cluster II (Figure 1A). A gene encoding a protein with peroxidase activity (PtpAffx.110158.2.A1_s_at) was up-regulated in both SS and SX stages and the protein was localized to the plasma membrane, indicating its potential role in H_2_O_2_ generation and PCD signalling. Two genes encoding peroxidase (PtpAffx.134095.1.A1_at) and an RCI3 (Ptp.7220.1.S1_s_at) were up-regulated in isolated xylem and localized in cell walls, indicating their possible role in the lignification of xylem’s cell walls after the protoplasts of the xylem cells have died. Two DEGs encoding peroxidase precursors and five DEGs encoding peroxidases were identified in developing pioneer roots. The expression of genes encoding peroxidase (PtpAffx.117730.1.S1_s_at) and peroxidase 27 (PtpAffx.224349.1.S1_s_at) was down-regulated in both roots with primary (PR) and secondary (SR) growth relative to their expression in root tips (RT), indicating that they may play a functional role in the early stages of xylem development. DEGs encoding cell-wall-localized peroxidase precursors (PtpAffx.249.187.S1_at and PtpAffx.224693.1.S1_s_at) were down-regulated in roots with secondary growth (SR). Additionally, two DEGs encoding peroxidases (PtpAffx.69019.1.A1_at and Ptp.7181.1.S1_at) were up-regulated in PR and SR, while another peroxidase (PtpAffx.225092.1.S1_at) DEG was up-regulated only in roots with secondary growth (Figure 1B). Four DEGs encoding proteins with peroxidase activity were identified in leaves. Two of them (PtpAffx.52399.1.S1_at and Ptp.5190.1.S1_at) were down-regulated during the LS2 stage. Another DEG (Ptp.7213.1.S1_s_at) was up-regulated in LS1 and LS2, while PtpAffx.205844.1.S1_at was not up-regulated until LS2 (Figure 1C). As many as six PXs precursor genes and seventeen peroxidase-encoding genes were observed to be differentially expressed in fine roots. Among them, two glutathione PXs, a glutaredoxin, a peroxiredoxin, and a TRX-dependant PX gene were up-regulated. In contrast, all six PX precursor DEGs and twelve PX DEGs were down-regulated in RS1 and RS2. Among the PXs, a gene encoding ascorbate peroxidase 3 was found to be down-regulated in fine roots (Figure 1D). 

Thioredoxins (TRXs) exhibit radical-scavenging activity and play a key role in redox signalling and oxidative stress responses. A gene encoding TRX was not significantly up-regulated until the SX stage in stems (Figure 1A). Additional genes encoding TRXs were identified in pioneer roots and two of them (PtpAffx.10732.1.S1_at and PtpAffx.10732.1.S1_a_at) were up-regulated in roots with primary and secondary growth, while a third (Ptp.5290.1.S1_at) was not up-regulated until pioneer roots had undergone secondary growth (SR) (Figure 1B). A single gene encoding TRX was identified in leaves, whose expression was up-regulated in both LS1 and LS2 stages (Figure 1C). Three DEGs encoding TRXs were identified in fine roots. One (PtpAffx.10732.1.S1_a_at) was up-regulated in RS1 and RS2, while the expression of the other two (PtpAffx.216528.1.S1_x_at and PtpAffx.18908.2.S1_a_at) was down-regulated in RS1 and/or RS2 (Figure 1D).

Amine oxidases (AOs), including polyamine oxidases (POAs), are involved in metabolic pathways that generate hydrogen peroxide [40,44]. Expression of an AO gene was down-regulated during both primary (PR) and secondary (SR) growth of pioneer roots, while a POA gene was up-regulated in roots with secondary growth (SR) (Figure 1B). Expression of a gene encoding AO was down-regulated in fine roots during the RS1 and RS2 stages. A similar pattern of expression was observed with four *mono-Cu oxidase precursor* and *mono-Cu oxidase-like precursor* genes, as well as for five genes encoding a multi-Cu oxidase type 1 protein (Figure 1D).

Hydrogen peroxide is scavenged during the oxidation of ascorbate (AsA) [45]. DEGs (PtpAffx.125778.1.A1_at and Ptp.5505.1.S1_at) encoding an L-galactose dehydrogenase, which functions in AsA biosynthesis, were down-regulated during primary and secondary growth of pioneer roots, indicating that ascorbate is synthesized to a greater extent at the beginning of root development. A gene encoding a cell-wall-localized ascorbate oxidase (AsO) precursor was down-regulated in roots with secondary growth, while another gene encoding an L-ascorbate oxygenase was up-regulated in roots with secondary growth (Figure 1B). A gene encoding galactose 1-phosphate phosphatase, which is involved in AsA biosynthesis, was not up-regulated in fine roots until the RS2 stage. Two genes encoding an L-galactose dehydrogenase, which is also involved in AsA biosynthesis, and one gene encoding an ascorbate oxidase precursor, were all down-regulated in fine roots in both RS1 and RS2 (Figure 1D).

Glutathione-S-transferase (GST) is an antioxidant enzyme catalysing conjugation of reduced glutathione during, e.g., detoxifying lipid hydroperoxides [46]. Six genes encoding GST were identified as DEGs in pioneer roots in our microarray analysis. Four grouped to Cluster II and were up-regulated in roots with primary growth (PtpAffx.133511.1.S1_at), secondary growth (Ptp.36.1.S1_s_at), or both (PtpAffx.224201.1.S1_at and PtpAffx.20731.1.A1_at). One gene encoding a glutathione-S-transferase TAU9 was down-regulated and one gene encoding a glutathione-S-transferase 30 was up-regulated in pioneer roots (Figure 1B). 

The expression of a gene related to singlet oxygen-mediated PCD was up-regulated in fine roots in the RS2 stage, suggesting its potential role in the later stages of fine root senescence (Figure 1D).

### 3.2. Superoxide Dismutase Activity Declines with Stem and Pioneer Root Development

**Figure 2:**
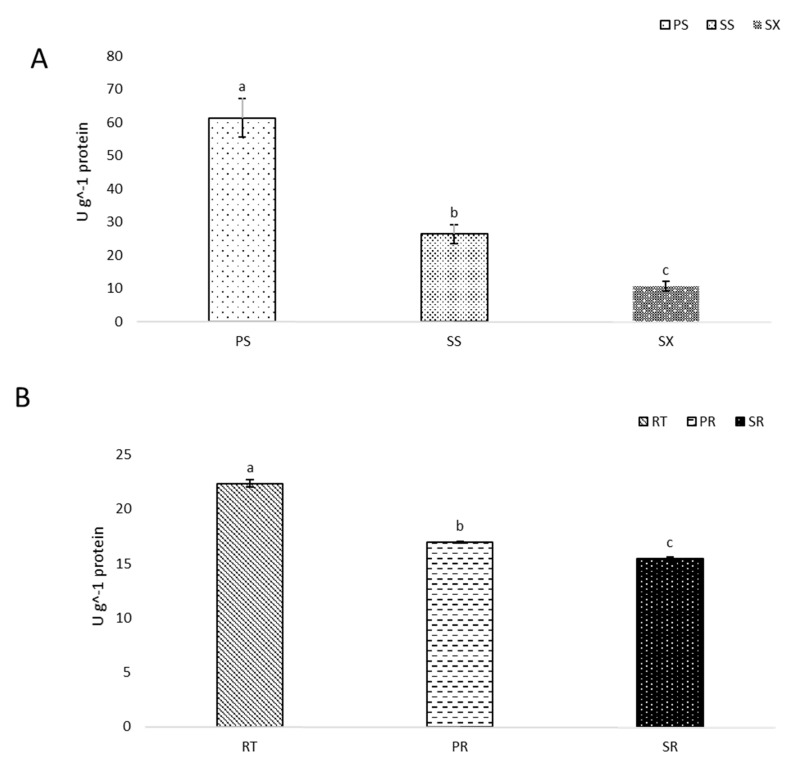
Superoxide dismutase (SOD) activity during the development of black cottonwood (*Populus trichocarpa*) stems (**A**) and pioneer roots (**B**): PS—stem apical meristem with stem primary growth, SS—stem with secondary growth, and SX—isolated secondary xylem. RT—root tip, PR—roots with primary growth, and SR—roots with secondary growth. Means designated by different letters indicate statistically significant differences according to a Tukey’s post hoc test (*p* < 0.05). Error bars indicate ± SE (standard error).

Similar to the expression of the Cu/Zn SOD gene, SOD activity in stems was highest in the youngest stem part (apical meristem with primary growth (PS)), and then significantly decreased down the stem as stems began to mature and exhibit secondary growth (SS), as well as in isolated samples of secondary xylem (SX) (Figure 2A).

SOD gene expression (PtpAffx.125778.1.A1_at and Ptp.5505.1.S1_at) was predominantly down-regulated in pioneer roots along with the progression from primary to secondary growth. The same pattern was observed for SOD enzyme activity, validating the pattern observed for SOD gene expression. Similar to stems, SOD activity in the pioneer roots was highest in the youngest portion of the root, the root tips with apical meristem (RT), and then declined with the progression from primary growth (PR) to secondary growth (SR) (Figure 2B). 

### 3.3. Identification and Localization of Superoxide

**Figure 3:**
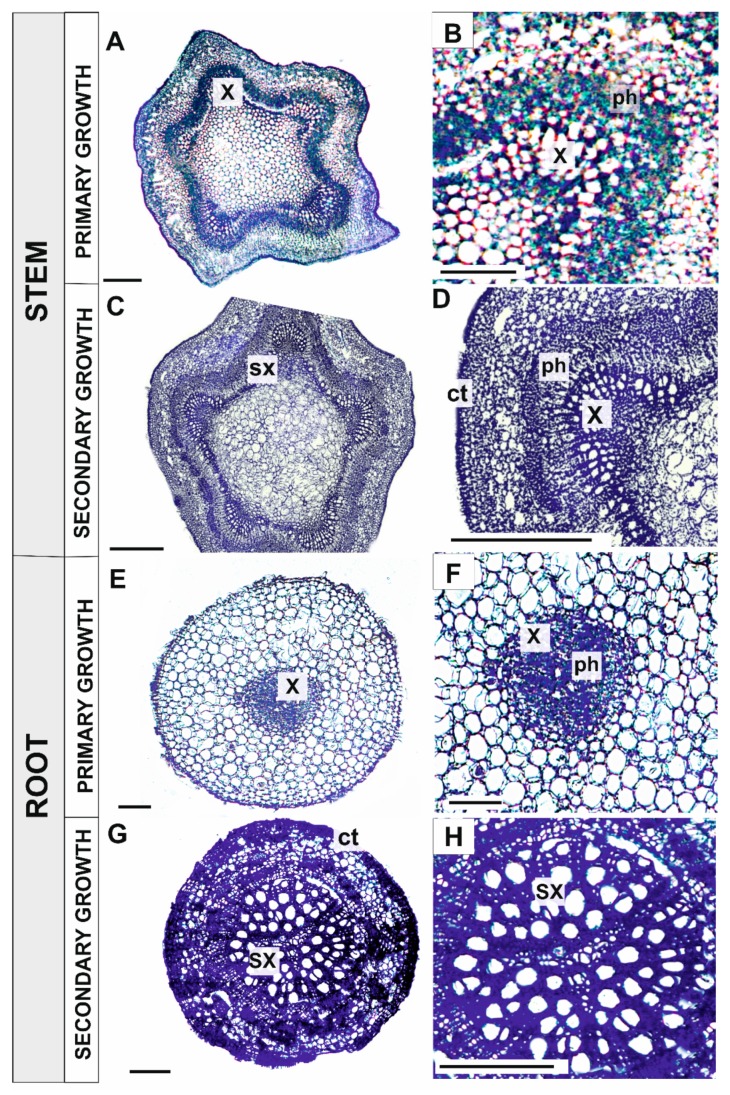
Localization of superoxide (dark blue dye) in stems of black cottonwood (*Populus trichocarpa*) with primary (**A,B**) and at the beginning of secondary growth (**C,D**), as well as in pioneer roots with primary growth (**E,F**) and secondary growth (**G,H**). (Abbreviations: x—primary xylem, sx—secondary xylem, ph—phloem, pf—phloem fibres, ct—cork (phellem)). Bars = 100 μm.

Nitrotetrazolium blue (NBT) staining was used to identify and determine the localization of superoxide in stems, pioneer roots, leaves, and fine roots of *Populus trichocarpa*. The highest accumulation of O_2_• ^–^ in stems with primary growth was observed in primary xylem (x) and phloem (ph) cells (Figure 3A,B). Superoxide accumulation was slightly lower in stem segments undergoing secondary growth than in stem segments with just primary growth. However, even in stems with secondary growth, superoxide was still observed in the cell walls of primary xylem (x) and developing phloem fibre (pf) cells (Figure 3C,D). The highest concentration of superoxide in pioneer roots with primary growth was observed in the vascular cylinder, primary xylem (x) and phloem (ph) cells (Figure 3E,F). In contrast, the strongest accumulation of O_2_•^–^ in pioneer roots with secondary growth occurred in the secondary xylem (sx), phloem fibre (pf), and cork (ct) cells (Figure 3G,H). 

**Figure 4:**
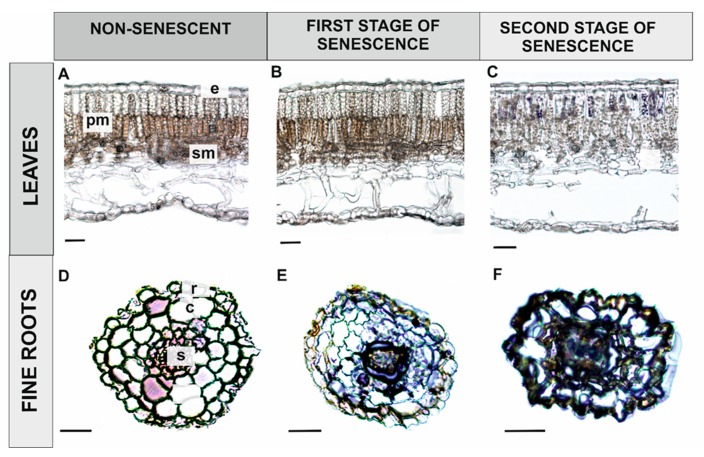
Localization of superoxide (dark blue dye) during leaf and fine root senescence in black cottonwood (*Populus trichocarpa*). Control leaf (**A**), first stage of leaf senescence (**B**), second stage of leaf senescence (**C**), fine root control (**D**), first stage of fine root senescence (**E**), second stage of fine root senescence (**F**). (Abbreviations: e—epidermis, pm—palisade mesophyll, sm—spongy mesophyll, r—rhizodermis, s—stele, c—cortex). Bars = 100 μm.

Superoxide accumulation was not observed in leaves until the second stage of senescence, when some O_2_•^–^ was evident in palisade parenchyma cells, especially at the second stage of leaf senescence (compare Figure 4A–C). No superoxide accumulation was observed in young, non-senescing fine roots, which served as a control (Figure 4D). The presence of O_2_•^–^ was observed in fine roots that were light brown in colour (RS1) in cortical parenchyma cells and inside the stele (Figure 4E). In older fine roots (RS2), superoxide was present in some cells within the stele, especially in the primary xylem, while cortical parenchyma cells were heavily degraded due to the ageing process, which created challenges for the observations of superoxide accumulation (Figure 4F). 

### 3.4. Identification and Localization of Hydrogen Peroxide

**Figure 5:**
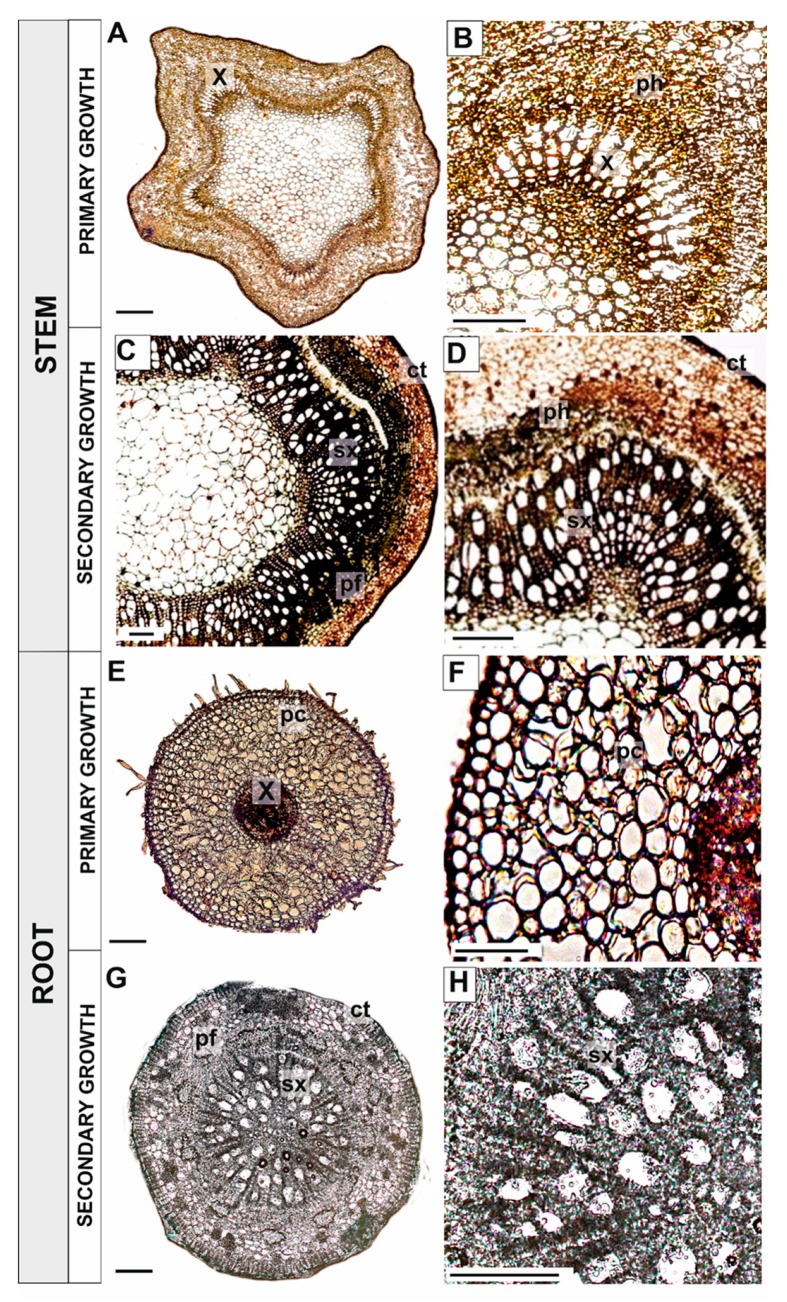
Localization of hydrogen peroxide (orange polymer) in the stems of black cottonwood (*Populus trichocarpa*) with primary growth (**A,B**), secondary growth (**C,D**), and in pioneer roots with primary growth (**E,F**), secondary growth (**G,H**). (Abbreviations: x—primary xylem, sx—secondary xylem, ph—phloem, pf—phloem fibres). Bars = 100 μm.

The use of 3-3’diaminobenzidine (DAB) staining was used to identify and determine the localization of hydrogen peroxide in developing stems and pioneer roots, as well as in the senescent leaves and fine roots of *Populus trichocarpa*. The highest accumulation of H_2_O_2_ in stems with primary growth was observed in primary xylem (x) and phloem (ph) cells (Figure 5A,B). Notably, the concentration of hydrogen peroxide in stem segments with secondary growth was significantly lower than in stems with just primary growth. The strongest accumulation of hydrogen peroxide in older stem segments occurred in phloem fibres (pf) and, to a lesser extent, in secondary xylem (sx) cells (Figure 5C,D). Hydrogen peroxide accumulated in the primary xylem (x) and phloem (ph) cells of pioneer roots with just primary growth (Figure 5E,F). The concentration of H_2_O_2_ in pioneer roots with secondary growth, however, was much lower, although hydrogen peroxide was detectable in the cell walls of secondary xylem (sx) and phloem fibre (pf) cells (Figure 5G,H).

A comparison of superoxide and hydrogen peroxide localization in developing stems and pioneer roots indicated that they are mostly localized in the same tissues. Specifically, they are localized in primary xylem and phloem cells in stems and pioneer roots with primary growth, as well as in secondary xylem and phloem fibre cells in stems and in pioneer roots with secondary growth.

**Figure 6:**
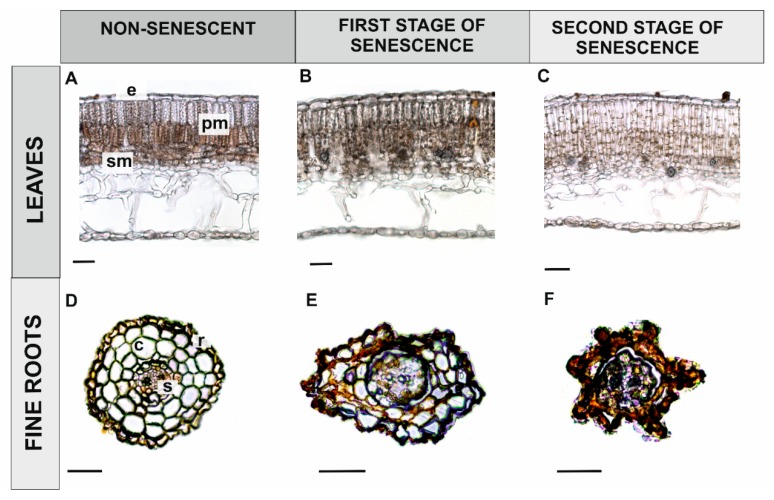
Localization of hydrogen peroxide (orange polymer) in senescing leaves and fine roots of black cottonwood (*Populus trichocarpa*). Control leaf (**A**), first stage of leaf senescence (**B**), second stage of leaf senescence (**C**), control fine root (**D**), first stage of fine root senescence (**E**), second stage of fine root senescence (**F**). (Abbreviations: e—epidermis, pm—palisade mesophyll, sm—spongy mesophyll, r—rhizodermis, s—stele, c—cortex). Bars = 100 μm.

Hydrogen peroxide accumulation was never observed in leaves at any of the studied stages of development (Figure 6A–C). Additionally, H_2_O_2_ was not observed in young, fine roots, or only observed in single cells within the stele (Figure 6D). In sections that were closer to the apical meristem, however, a significant accumulation of hydrogen peroxide was observed, especially in the endodermis and cortical parenchyma cells (not shown). In fine roots (RS1) that were light brown in colour, the accumulation of H_2_O_2_ was primarily observed in cortical parenchyma cells with altered structure, a symptom of senescence, and also within the stele (Figure 6E). Most cortical parenchyma cells were completely degraded in the oldest fine roots (RS2), however, hydrogen peroxide accumulation was still visible there, as well as within stele cells (Figure 6F).

Superoxide and hydrogen peroxide exhibited the same tissue localization in light brown fine roots (RS1), namely, in cortical parenchyma cells and in cells within the stele. These compounds were also localized to the same cells within the stele in the oldest fine roots (RS2).

## 4. Discussion

Reactive oxygen species play a significant functional role in plant developmental processes, with their presence often regulating the induction of many developmental or physiological processes, including seed germination, histogenesis, and root gravitropism [4]. ROS often act as signalling molecules in living organisms [47], triggering or regulating the onset of ageing, programmed cell death, and response to biotic and abiotic stress, including pathogens [21]. The generation of ROS involves a cascade of consecutive events which are tightly regulated by antioxidant enzymes that act as catalysts for specific reactions [47,48]. While the role of ROS and antioxidants in the lignification of tracheary elements in cell cultures and their role in xylogenesis in developing stems are well known, information is lacking on their role in developmental processes in underground organs, such as pioneer roots. Only a few recent studies have compared pioneer root development to the ontogeny of stems [31,32]. In these studies, similarities were noted in cell wall development and the PCD of xylem cells, as well as organ-specific mechanisms. The process of senescence has also been widely studied in leaves and petals, including the role of ROS in the senescence process. Again, however, significantly less is known about the regulation of senescence in underground organs, such as fine absorptive roots, compared to the senescence in aboveground organs. Recent studies have indicated, however, that senescence processes in above and belowground organs exhibit many similarities, especially regarding ultrastructural changes and the activation of autophagy-related mechanisms [18]. However, organ-specific modes of action were observed during the remobilization and resorption of nutrients during the seasonal senescence of leaves and fine roots [49]. Those initial studies made it very clear that there was a significant knowledge gap pertaining to the physiological and molecular role of ROS in the development and senescence of belowground organs. 

### 4.1. ROS Are Involved in Xylem Development

The cell wall development process in higher plants is closely associated with the presence of ROS [50]. Hydrogen peroxide is a substrate for the synthesis of lignin, and elevated levels of superoxide anion radicals, while not lethal, function as a signal to initiate the lignification process [51]. The incrustation of lignin into cell walls greatly increases their mechanical strength and makes them more resistant to pathogen attack and penetration [21]. Cell wall lignification also enables conducting xylem cells (tracheids and vessel elements) to transport water containing dissolved mineral salts. Xylogenesis, the differentiation and maturation of tracheary elements, is one of the processes in which programmed cell death has been well documented [12,14,31,52]. PCD during xylogenesis occurs concomitantly with the deposition of the secondary cell wall. The proper course of this process is partly regulated by the action of antioxidant enzymes, such as peroxidase or superoxide dismutase. 

Hydrogen peroxide, which spontaneously forms after the generation of superoxide (O_2_•^–^) by RBOHs, is used by xylem peroxidases for the polymerization of cinnamyl alcohols [39]. RBOHs are involved in both long-distance ROS signalling and a localized ROS burst [40]. As a result, RBOHs may act as a focal point in ROS signalling [2,53] by integrating ROS signals with MAPKs [54,55]. In the present study, genes encoding RBOHs were up-regulated in stems with secondary growth (SS) and isolated secondary xylem (SX), which suggests their role in the lignification of xylem both prior to and after the death of a xylem cell. In contrast, *RBOHD* was down-regulated in more developed pioneer roots, which may indicate that *RBOHD* may only play a role during the early stages of xylogenesis in pioneer roots.

Ogawa et al. [56] indicated that Cu/Zn SOD may co-localize with RBOH and cooperatively interact with SOD during the transformation of superoxide, generated by RBOH, into hydrogen peroxide. Cu/Zn SOD was demonstrated to be involved in the regulation of H_2_O_2_ during SCW formation in tracheary elements of *Zinnia elegans* [50]. High levels of SOD activity in cell cultures [50] and tobacco plants [8] have been shown to determine the correct functioning of the last stage of xylogenesis (the formation of secondary cell walls), and to also initiate PCD by producing hydrogen peroxide, which acts as a signalling molecule to start the PCD process. Our analysis of pioneer root samples indicated that *Cu/Zn SOD1* was up-regulated in samples exhibiting both primary (PR) and secondary (SR) development, suggesting a potential role for *Cu/Zn SOD1* in the generation of H_2_O_2_ in *P. trichocarpa*, which could be used for cell wall lignification. 

Two main groups of peroxidases can be distinguished: the peroxidase S group, with the ability to oxidize sinapyl alcohol, and the peroxidase G group, which includes the remaining peroxidase enzymes. Peroxidases actively participate in cell wall loosening during polysaccharide cleavage by cellulases [57,58] and in the generation of hydroxyl radicals (OH•), which break covalent bonds in cell wall polymers, such as pectins and xyloglucans [59]. They also play a role in cell wall stiffening during lignification [60,61]. Peroxidases are one of the enzymes that determine the proper course of xylogenesis by polymerizing sinapyl alcohol to lignin in the presence of hydrogen peroxide [42] (Marjamaa et al., 2009). The role of peroxidases in the lignification process has been thoroughly described in studies conducted in *Zinnia elegans* cell cultures [62], where a peak in ZePrx activity was highly correlated with the formation of tracheary elements and where tracheary development was shown to be dependent on plant hormones, hydrogen peroxide, and nitric oxide. El Mansouri et al. [63] reported that an increase in alkaline peroxidase gene expression was responsible for increased lignin synthesis in tomato (*Solanum lycopersicum*). Notably, a decrease in the expression of a gene encoding an anionic PrxA3a in stems of hybrid aspen (*Populus sieboldii* x *P. gradidentata*) was associated with a 20% decrease in lignin biosynthesis [64]. In addition, *Arabidopsis thaliana* mutants, in which the gene encoding peroxidase 72 was inactivated, exhibited fewer syringyl subunits and a reduced level of lignification compared to control plants [65]. De Pinto et al. [8] reported a decrease in the expression of peroxidase-coding genes in samples of tobacco (*Nicotiana tabacum*) with secondary development, including well-developed secondary xylem. Notably, they demonstrated that PCD leads to a decrease in the expression of genes encoding ascorbate and glutathione peroxidase. These findings suggest that changes in the expression of peroxidase genes may represent the first signal to initiate PCD during xylogenesis. Studies on peroxidase activity during the occurrence of programmed cell death during xylogenesis have shown a decrease in peroxidase activity, which is directly related to a decrease in antioxidant defence and leads to the generation of a PCD-induced oxidative burst [8]. In the current study, however, expression of all the *PX*s genes identified in stems were up-regulated. Increased expression of a plasma membrane-localized peroxidase (PtpAffx.110158.2.A1_s_at) indicates its potential role in H_2_O_2_ production and PCD signalling in stems. *RCI3* gene expression is responsible for programmed cell death in plants exposed to low-temperature stress [66]. The increased expression of this gene, which encodes a cell-wall localized protein, in isolated secondary xylem (SX) cells from stems, indicates that *RCI3* may also be involved in PCD or/and lignification of xylem cells in *Populus trichocarpa* during xylogenesis. Our previous study [32] demonstrated that the expression of genes encoding enzymes involved in lignification increased during stem development and grouped in Cluster II. Similar results for all genes encoding PXs were obtained in the present study. Therefore, the up-regulated expression of cell-wall-localized peroxidases in isolated xylem suggests their role in the post-mortem lignification of stem xylem cells. Two patterns can be observed in pioneer roots for genes encoding peroxidases and peroxidase precursors. In one pattern, *PX* expression decreased as roots developed, indicating their involvement in early stages of xylem development, such as cell wall loosening. In the second pattern, peroxidase precursors are down-regulated with development, possibly because the lignification process in xylem is nearing completion. As a result, peroxidase precursors are no longer needed. The up-regulation of peroxidase gene expression, however, indicates their involvement in primary and secondary cell wall development and lignification, possibly even after the xylem cells undergo PCD.

The role of peroxidases in the process of xylem formation involves the presence of hydrogen peroxide, which is a substrate for peroxidases during the polymerization of sinapyl alcohol to lignin [62]. The accumulation of hydrogen peroxide observed in young growing stems and pioneer roots (Figure 4) in our study confirms this hypothesis. The decrease in peroxidase activity in pioneer roots during xylogenesis enables the correct process of PCD to occur as the accumulation of hydrogen peroxide induces oxidative stress, which acts as a signal to initiate developmental processes such as cell wall lignification and PCD [4]. Regarding xylogenesis, the accumulation of superoxide anion radicals that was observed in the primary xylem and secondary xylem of stems and pioneer roots (Figure 3) may act as a signal to initiate the degradative processes that occur during PCD, as well as cell wall lignification.

Amine oxidases (AOs), including polyamine oxidases (POAs), represent additional enzymes that are involved in metabolic pathways where hydrogen peroxide is generated [40]. AOs play a role in the generation of H_2_O_2_ used during peroxidase-mediated cell wall cross-linking [67]. AOs are also associated with PCD during xylogenesis by their generation of H_2_O_2_ [67,68]. Down-regulation of *AO* expression and increasing expression of *POA* in pioneer roots indicates that POA rather than AOs is involved in SCW modification and PCD during xylogenesis. Since AOs are involved in the generation of H_2_O_2,_ used to signal the initiation of PCD and in the enzymatic process of cell wall lignification by peroxidases, it is possible that the down-regulation of genes encoding AO and Cu-oxidases in fine roots indicates that these genes are only involved in the early stages of xylogenesis and PCD in young fine roots.

Ascorbate is also involved in ROS signaling and scavenging [45,69]. In the current study, AsA was synthesized more intensively at the beginning of pioneer root development, suggesting that ROS are scavenged in young roots. In contrast, a gene encoding a cell-wall-localized ascorbate oxidase (AsO) precursor was down-regulated in roots with secondary growth. Since AsO is associated with rapidly expanding cell walls [45], our results on AsO may indicate that it only plays a role in xylem cell development during the primary development of pioneer roots. Another L-ascorbate oxygenase expressed in pioneer roots with secondary growth, however, appears to be involved in H_2_O_2_ scavenging at the end of the lignification process when hydrogen peroxide is no longer needed.

### 4.2. ROS Play an Important Role in Developmental Process Involving PCD

RBOHD is the key enzyme responsible for the rapid production of apoplastic ROS in response to pathogen invasion [70]. Based on the study by van Aken and van Breuseyem [71], it appears that the role of NADPH oxidase in senescence is marginal. Up-regulation of a gene encoding RBOHD was observed in our study in leaves, while up-regulation of NADP oxidase was observed in fine roots, indicating that they have a role in generating the H_2_O_2_ that serves as a signal molecule in the induction of PCD by an ROS burst. 

High levels of SOD activity are known to initiate programmed cell death by producing hydrogen peroxide, which functions as a signalling molecule. Over the course of PCD, SOD activity increases several-fold, leading to a significant increase in the accumulation of hydrogen peroxide in cells, and finally resulting in an oxidative burst that initiates PCD [8,49]. The results of the current study suggest that SOD was potentially responsible for generating H_2_O_2_ during early stem development, which acted as a signalling molecule that induced PCD. Our results also indicated, however, that SOD is not involved in the senescence process in leaves but does play a role in the senescence of fine roots, where Cu/Zn SOD may generate sufficient levels of H_2_O_2_ to induce PCD by acting as a signal molecule.

*EDS1* (*enhanced disease susceptibility 1*) plays a crucial role in the production and accumulation of H_2_O_2_ and is also involved in initiating the cell death signalling pathway in response to biotic and abiotic stresses [41]. Increasing levels of *ESD1* expression along developing stems may lead to the accumulation of hydrogen peroxide which may play a role in PCD signalling during xylogenesis. In fact, our results indicate that *ESD1* may be a component of the cell death signalling pathway in response to stress as well as primary and secondary xylem development in stems.

In addition to their role in cell wall lignification, peroxidases function as antioxidants by inactivating ROS, thus defending cells against the injurious effect of detrimental concentrations of ROS [62,72]. The expression profiles of genes encoding peroxidases in leaves suggest their involvement in ROS scavenging in younger leaves, since the expression of *PX*s was elevated in younger leaves. Additionally, they are also related to the generation of H_2_O_2_ to the PCD signalling pathway in senescing leaves, since *PX*s were strongly expressed during the later stages of senescence. In contrast, few *PX* genes were up-regulated during the ageing of fine roots, which may imply that these *PX* genes are involved in H_2_O_2_ generation and PCD signalling. The majority of *PX* genes were down-regulated in senescing fine roots, which would have resulted in reduced levels of ROS scavenging. Consequently, this would lead to an accumulation of ROS, resulting in an oxidative burst and finally cell death. According to Zimmermann et al. [72], down-regulation of *ascorbate peroxidase 1* and increased levels of H_2_O_2_ act as a signal to induce the expression of senescence-associated TFs and genes. Therefore, it is possible that the decreased expression of *ascorbate peroxidase 3* observed in the current study, combined with an elevated level of hydrogen peroxide (Figure 6E,F), may also be involved in inducing senescence in fine roots. 

Thioredoxins (TRXs) are involved in apoptosis signalling pathways in animals [73,74]. Thus, the increased expression of *TRX*s in stems and pioneer roots during development may indicate their potential function as a signal molecule in PCD during xylogenesis. Genes encoding TRXs were also up-regulated in older leaves and fine roots, suggesting the involvement of TRX in the PCD signalling pathway associated with senescence. TRXs, however, appear to also function as ROS-scavengers in young, fine roots.

H_2_O_2_ is scavenged during the oxidation of ascorbate [45]. The majority of genes involved in ascorbate biosynthesis were down-regulated in senescing fine roots, indicating that hydrogen peroxide scavenging decreases with age, resulting in an accumulation of H_2_O_2_. Consequently, this may then lead to an oxidative burst and PCD. Therefore, we speculate that decreased biosynthesis of AsA initiates PCD in ageing fine roots that are undergoing senescence. 

Smart et al. [75] observed that glutathione-S-transferase transcripts increase during leaf senescence. In the current study, the majority of genes encoding GST were up-regulated in pioneer roots with primary and/or secondary growth (Figure 1A). These data suggest that GST is also involved in the PCD of xylem cells, as well as the PCD that occurs during the senescence process in leaves. Apart from the up-regulation of *GST30* in leaves that may have a role in PCD signalling during leaf senescence, we also identified the down-regulation of *GST TAU9* which may be involved in ROS-scavenging in young leaves. 

Singlet oxygen injures cell membranes and other components, which ultimately leads to cell death [76]. In addition, it also functions as a signal molecule by activating senescence-associated genes [77,78]. Singlet oxygen was reported to play a crucial role in senescence-associated oxidative stress in sage (*Salvia* *officinalis*) chloroplasts [79]. Increased level of lipid peroxidation caused by ^1^O_2_ leads to cell death [80,81]. Therefore, the up-regulation of a gene related to singlet oxygen-mediated PCD in the RS2 stage of senescing fine roots (Figure 1D) suggests that it may also play a role in the late stages of fine root senescence in *Populus trichocarpa*.

In addition to its role as a signalling molecule, hydrogen peroxide is also involved in the final stages of senescence by contributing to cell degradation [82,83]. H_2_O_2_ is an important element of the network regulating the course of PCD [10]. During the process of bolting and flowering, H_2_O_2_ levels were observed to increase, while ascorbate PX1 activity decreased [82]. In this case, it is plausible that H_2_O_2_ may act as a signal to induce the expression of senescence-associated TFs and genes [21]. The results of the current study indicate that hydrogen peroxide is not involved in the production of an oxidative burst and cell degradation during leaf senescence. It is possible, however, that a small amount of H_2_O_2_, too low to be detected by as simple method as DAB staining, does act as a signal molecule. In contrast, increasing accumulation of H_2_O_2_ (Figure 6E,F) and a decreasing level of expression of PX genes were observed in fine roots. Wojciechowska et al. [18] reported that ATG8 (AuTophaGy-related) protein, a key component in the formation of autophagosomes, was localized in fine roots in the same tissues that hydrogen peroxide and superoxide radicals were localized in the present study. Therefore, we suggest that the localization of ROS and ATG8 in the same tissues strongly supports their contribution to cell degradation during the senescence of fine roots. 

We also observed the localization of superoxide radicals in the chloroplasts of palisade parenchyma cells in the oldest leaves (Figure 4C). In accordance with these observations, Wojciechowska et al. [18] also reported the accumulation of ATG8 protein in the same cells. Chloroplast degradation occurs during the later stages of PCD [84] and chloroplasts are a primary source of ROS in plants. Therefore, palisade parenchyma cells in senescing leaves of *Populus trichocarpa* may be targeted for degradation by the process of chlorophagy [85,86].

## 5. Conclusions

**Figure 7:**
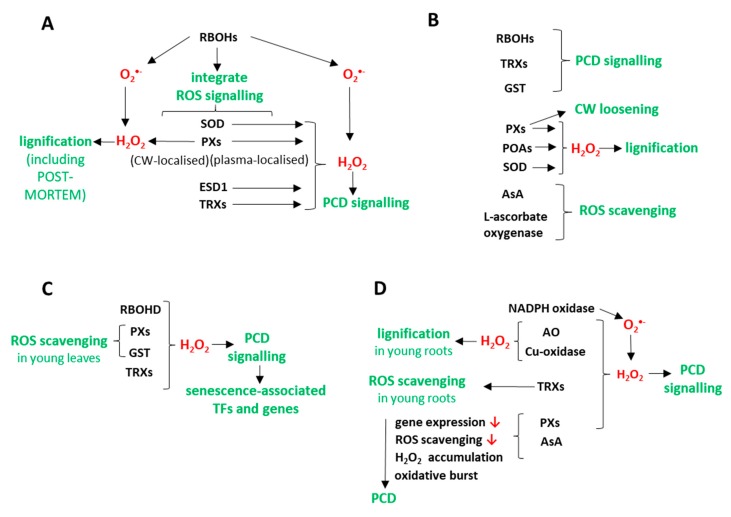
Simplified models describing the major changes in ROS metabolism in stems (**A**) and pioneer roots (**B**) during their primary and secondary growth, and in senescing leaves (**C**) and fine roots (**D**) of *Populus trichocarpa*. The downward red arrows show decreased features. (Abbreviations: RBOHs - respiratory burst oxidase homologs, O_2_•^–^—superoxide, ROS—reactive oxygen species, SOD—superoxide dismutase, PXs—peroxidases, CW—cell wall, H_2_O_2_—hydrogen peroxide, ESD1—enhanced disease susceptibility 1, TRXs—thioredoxins, PCD—programmed cell death, GST—glutathione-S-transferase, POAs—polyamine oxidases, AsA—ascorbate, TFs—transcription factors, AO—amine oxidases).

ROS and ROS-related enzymes exhibit three main functions during developmental processes in *Populus trichocarpa.* First, excessive levels of ROS result in damage to cell components in younger organs; a process which is minimized through ROS-scavenging by antioxidants (Figure 7B–D). Second, the generation of hydrogen peroxide occurs as an important part of cell wall lignification in the developing xylem of stems and pioneer roots, as well as in young fine roots (Figure 7A,B,D). Third, they are also involved in PCD signalling, which is associated with both xylem development in stems and pioneer roots, and the senescence of leaves and fine roots (Figure 7A–D). Many ROS-related enzymes participate in generating ROS and in cell death signalling. This may superficially seem like self-sabotage, or a system error causing the production of unwanted “enemies” (ROS molecules). In fact, however, ROS generation is a highly regulated and programmed strategy where these “allies” (ROS molecules) enable the proper development of cells and tissues, including xylem development and the natural cycle of senescence when valuable substances are remobilized and resorbed. 

## Figures and Tables

**Table 1:** Nomenclature describing the sampling of *Populus trichocarpa* stems, pioneer roots, leaves and fine roots.

Organ	Abbreviation	Characteristic Features
Stem	PS	apical meristem with primary growth
	SS	secondary growth
	SX	isolated secondary xylem
Pioneer Roots	RT	root tip with apical meristem
	PR	primary growth
	SR	secondary growth
Leaves	LC	control green leaves without senescence symptoms
	LS1	first stage of senescence: yellowing leaves in which chlorophyll level had decreased by approximately 40%
	LS2	second stage of senescence: yellow leaves in which chlorophyll level had decreased by approximately 60%
Fine Roots	RC	control white roots without senescence symptoms
	RS1	first stage of senescence: roots which had changed in colour from white to brown
	RS2	second stage of senescence: roots which had changed in colour from brown to dark brown or almost black, shrinkage was also visible in most fine roots
